# Measuring the impacts of maternal child marriage and maternal intimate partner violence and the moderating effects of proximity to conflict on stunting among children under 5 in post-conflict Sri Lanka

**DOI:** 10.1016/j.ssmph.2022.101074

**Published:** 2022-03-22

**Authors:** Ruvani W. Fonseka, Lotus McDougal, Anita Raj, Elizabeth Reed, Rebecka Lundgren, Lianne Urada, Jay G. Silverman

**Affiliations:** a Center on Gender Equity and Health, University of California San Diego School of Medicine, 9500 Gilman Drive, La Jolla, CA, 92093, USA; b San Diego State University/University of California, San Diego Joint Doctoral Program in Public Health, 9500 Gilman Dr, La Jolla, CA, 92093, USA; c San Diego State University School of Public Health, 5500 Campanile Drive, San Diego, CA, 92182, USA; d San Diego State University School of Social Work, 5500 Campanile Drive, San Diego, CA, 92182, USA; e San José State University School of Social Work, 1 Washington Square, San Jose, CA 95112, USA

**Keywords:** Sri Lanka, Girl child marriage, Intimate partner violence (IPV), Post-conflict, Stunting

## Abstract

This study aimed to understand whether maternal child marriage and past year intimate partner violence (IPV) impact stunting among Sri Lankan children under 5 years old, and, secondarily, whether proximity to conflict is associated with stunting. Additionally, we assessed whether proximity to conflict moderates the relationships between maternal child marriage and past year IPV (sexual, physical, and emotional). We tested these questions using logistic regression analyses of the 2016 Sri Lankan Demographic and Health Survey (n = 4941 mother-child dyads). In country-wide adjusted analyses, we did not find associations between maternal child marriage or IPV and stunting (p > 0.05). Children in districts proximal and central to conflict were significantly less likely to be stunted compared to children in districts distal to conflict (proximal adjusted odds ratio/aOR: 0.43, 95% confidence interval/CI: 0.22–0.82; central aOR: 0.53, CI: 0.29–0.98). We found significant interaction effects on stunting between proximity to conflict and both sexual and emotional IPV, which we further explored in stratified analyses. In districts distal to conflict, maternal sexual IPV was significantly associated with increased odds of stunting (aOR: 2.71, CI: 1.16–6.35), and in districts central to conflict, maternal emotional IPV was significantly associated with increased odds of stunting (aOR: 1.80, CI: 1.13–2.89). Maternal emotional IPV was significantly associated with decreased odds of stunting in districts proximal to conflict (aOR: 0.42, CI: 0.18–0.96). Maternal child marriage and physical IPV were not associated with stunting in Sri Lanka. Variations in associations between maternal IPV and stunting across Sri Lanka may reflect the lasting and differential impact of conflict, as well as differential humanitarian responses which may have improved child nutrition practices and resources in districts central and proximal to conflict. Policies and programs addressing stunting in Sri Lanka should consider the role of maternal IPV as well as community-level variations based on proximity to conflict.

## Highlights

• Across Sri Lanka, stunting is reduced in areas proximal and central to conflict.
• Proximity to conflict moderates the impact of sexual and emotional IPV on stunting.
• Children's vulnerability to stunting and IPV varies by proximity to conflict.
• Humanitarian aid may have decreased stunting in areas most impacted by conflict.

## 1 Introduction

Stunting, or low height-for-age, is the most prevalent form of child malnutrition in the world, and is experienced by 161 million children aged 0–5 years (de Onis & Branca, 2016). Stunting has been shown to be associated with increased childhood morbidity and mortality, reduced cognitive function, and elevated risk of chronic disease in adulthood, and addressing it has been identified by the World Health Organization as a major global health priority (de Onis & Branca, 2016; World Health Organization, 2015). Many socioeconomic risk factors for stunting have been identified in the research literature, such as wealth, feeding practices, household food insecurity, and lower levels of parental education (Rannan-Eliya et al., 2013; Wondimagegn, 2014). In addition to socioeconomic factors, some studies have identified associations between stunting and maternal characteristics such as height and ethnicity that suggest possible genetic drivers of stunting in children, particularly in communities with low average heights (de Onis & Branca, 2016; Rannan-Eliya et al., 2013). The causes of stunting for any child are numerous and context-specific and have yet to be fully understood.

Two maternal gender inequities that appear to be associated with stunting in different settings are girl child marriage and intimate partner violence (IPV). Maternal child marriage has been found in multiple contexts to be associated with increased child malnutrition and infant mortality (Raj et al., 2010, 2014). It has been specifically linked to increased odds of stunting across multiple countries in Sub-Saharan Africa (Efevbera et al., 2017), potentially through the mechanisms of early childbearing and reductions in maternal education, which can impact maternal behaviors affecting child health, such as choosing to breastfeed, immunize, and educate children. Maternal experience of IPV has also been found to be associated with increased likelihood of stunting in settings in Africa and South Asia (Sobkoviak et al., 2012; Ziaei et al., 2014), possibly as a result of increased household stress and negative impacts on nutritional practices, such as breastfeeding (Misch & Yount, 2014). South Asia has the highest prevalence of girl child marriage in the world at 46 percent, and a 41 percent prevalence of IPV (Loaiza Sr. & Wong, 2012; World Health Organization, 2013), further incentivizing study in the region on these factors’ influence on child stunting.

Proximity to conflict is another factor that may impact stunting and could plausibly moderate the relationships between maternal child marriage, maternal IPV, and stunting. Studies in multiple conflict-affected countries in Sub-Saharan Africa have found armed conflict to be associated with negative child health outcomes, increased child mortality, and increased likelihood of stunting (Boerma et al., 2019; Minoiu & Shemyakina, 2014; O'hare & Southall, 2007). Researchers have found that, by increasing social and economic vulnerabilities, conflict can increase the likelihood of child marriage in war-affected communities (Gottschalk, 2007; Mourtada et al., 2017; Raj, 2010). Living in a post-conflict setting has also been shown to increase the likelihood of recent IPV experience in multiple countries (Bandara et al., 2021; Kelly et al., 2018). Long-term exposure to armed conflict can create changes in interpersonal interactions, social practices, and access to resources, which may explain the associations between proximity to conflict and child marriage, IPV, and stunting. It is also plausible that proximity to conflict may alter the mechanisms by which maternal child marriage and IPV influence child stunting, thereby moderating any impacts of maternal child marriage and IPV on stunting in post-conflict settings.

In Sri Lanka, which emerged from a prolonged conflict in 2009, post-conflict associations between maternal child marriage, IPV, proximity to conflict, and stunting remain poorly understood. The conflict adversely affected health care infrastructure in the Northern and Eastern provinces of the country, and created socioeconomic barriers to thriving that have persisted for many years post-conflict (Jayasekara & Schultz, 2007; McCall, 2016). Researchers in Sri Lanka have identified low household wealth, short maternal height, and low birthweight as associated with stunting in children age 0 to 5 (Rannan-Eliya et al., 2013). While the post-conflict prevalence of stunting ranges from 26% to 44% in areas proximal and central to the conflict in Sri Lanka (Kandeepan et al., 2016; Naotunna et al., 2017), no national studies have assessed the impact of proximity to conflict on childhood experience of stunting. Studies of child marriage conducted during and after the conflict have identified increased rates of child marriage in areas central and proximal to conflict compared to those distal from the conflict (FOKUS WOMEN, 2015; Kottegoda et al., 2008). A recent Sri Lankan study found that IPV was most likely to occur in areas central to conflict and in the context of socioeconomic disadvantage (Bandara et al., 2021). The co-occurrence of socioeconomic hardship and conflict in Sri Lanka creates a need for multivariable analyses that assess the independent contributions of maternal child marriage and IPV to stunting while controlling for other associated factors. Additionally, it is possible that the prolonged conflict has altered relationships between child marriage, IPV and stunting by changing the environment in which they occur – assessing for moderation can help uncover whether these relationships differ by proximity to conflict.

This study aims to understand whether maternal child marriage and maternal IPV are associated with stunting in Sri Lankan children under 5 years old, adjusting for proximity to conflict. We secondarily examine effects of proximity to conflict on stunting in Sri Lanka. The study also seeks to identify if proximity to conflict acts as a moderator for associations of maternal child marriage and IPV with stunting. The findings from this study can expand our understanding of linkages between women's lived experiences and the health of their children, as well as additional vulnerabilities or challenges that may be faced by families in post-conflict settings.

## 2 Materials and methods

### 2.1 Data source: 2016 Sri Lankan Demographic and health survey

This study used data from the 2016 Sri Lankan Demographic and Health Survey (DHS), which collected data on child and maternal health outcomes, domestic violence experience, reproductive health, and the economic engagement and agency of women in Sri Lanka (N = 27,210 households, 18,510 women aged 15–49 years, and 8146 children aged 0–59 months). The 2016 survey was the first DHS in Sri Lanka to be conducted using a nationally-representative sample of households; all previous DHS data collection in Sri Lanka occurred during the 30-year civil war and excluded portions of the Northern and Eastern provinces, which had been claimed as a separate state by the Liberation Tigers of Tamil Eelam (LTTE) organization (Sengupta, 2009).

In addition to a general health survey administered to every eligible woman in each household, a domestic violence module was administered to one woman in each household (n = 16,629). The 2016 DHS was the first to ask questions on experience of past year intimate partner violence (IPV) in Sri Lanka and, to-date, only two peer-reviewed studies have been published using this IPV data, neither of which also examine child health (Fonseka et al., 2022a, 2022b). Following the World Health Organization's guidelines for the ethical collection of information on domestic violence, one eligible woman per household was randomly selected for the module, which was not implemented if privacy could not be obtained (Ellsberg & Heise, 2005). Each domestic violence module respondent was read an additional consent statement at the start of the module, informing her that the questions could be personal and reassuring her of the confidentiality of her responses. In the 2016 DHS, women taking part in the domestic violence module were asked nine questions about their experiences of intimate partner violence in the previous 12 months (Department of Census and Statistics, 2017). In addition to asking women about their own health, the enumerators collected health and anthropometric data for all children under the age of five years in each household (n = 8146). These children's data can be linked to that of their mothers, allowing for analyses of relationships between maternal characteristics and child health.

This study combines data on household-level characteristics, women's experiences of child marriage and IPV, and under-5 child health. The total number of child-mother dyads available in the DHS dataset was 7908. Our study sample was limited to singleton-birth children (Kannaujiya et al., 2020) whose mothers were currently living with an intimate partner, had answered the domestic violence module of the DHS, and were age 18 and above. This restriction to mothers over 18 follows a convention in girl child marriage research to censor participants under age 18 who might marry after the survey's completion to avoid erroneously counting as not married as children girls who eventually are (Loaiza Sr. & Wong, 2012). After excluding participants who did not fit the study criteria, the sample included in this study comprised of 4941 child-mother dyads. Ethical approval for this research was obtained from the University of California, San Diego Institutional Review Board (Project number #191418XX).

### 2.2 Variables of interest

#### 2.2.1 Dependent variable: Stunting

As the dependent variable, we examined stunting of children aged 0–59 months. Using the WHO definition of stunting, we defined children as experiencing stunting if their height-for-age measurement was more than two standard deviations below the median height-for-age of the WHO Child Growth Standards in their age and sex category (World Health Organization, 2015).

#### 2.2.2 Independent variables: maternal child marriage and intimate partner violence

We examined four variables as independent variables of interest, to account for the separate and overlapping effects of different experiences. The four binary independent variables we considered are listed below:

1. Maternal child marriage: the mother married or cohabited with a male partner before age 18; yes or no.
2. Maternal past year sexual IPV: the mother had been forced to have sex by a partner in the last 12 months; yes or no.
3. Maternal past year physical IPV: the mother had experienced at least one of six types of physical intimate partner violence in the last 12 months: 1) slapping or beating with a hand, 2) pushing or shoving, 3) strangulation, 4) dragging or pulling, 5) beating with an object, or 6) burned; yes, or no.
4. Maternal past year emotional IPV: the mother had experienced at least one of two incidents in the last 12 months, either being belittled/offended or prevented from leaving home by a partner; yes or no.

We considered maternal past year physical, sexual, and emotional IPV as separate independent variables to allow estimation of the independent effects of each form of IPV. We calculated Spearman's rho correlation estimates across all three IPV variables. All IPV correlation estimates were below 0.5, justifying retaining them as independent variables.

#### 2.2.3 Moderating variable: proximity to conflict

**Fig. 1:**
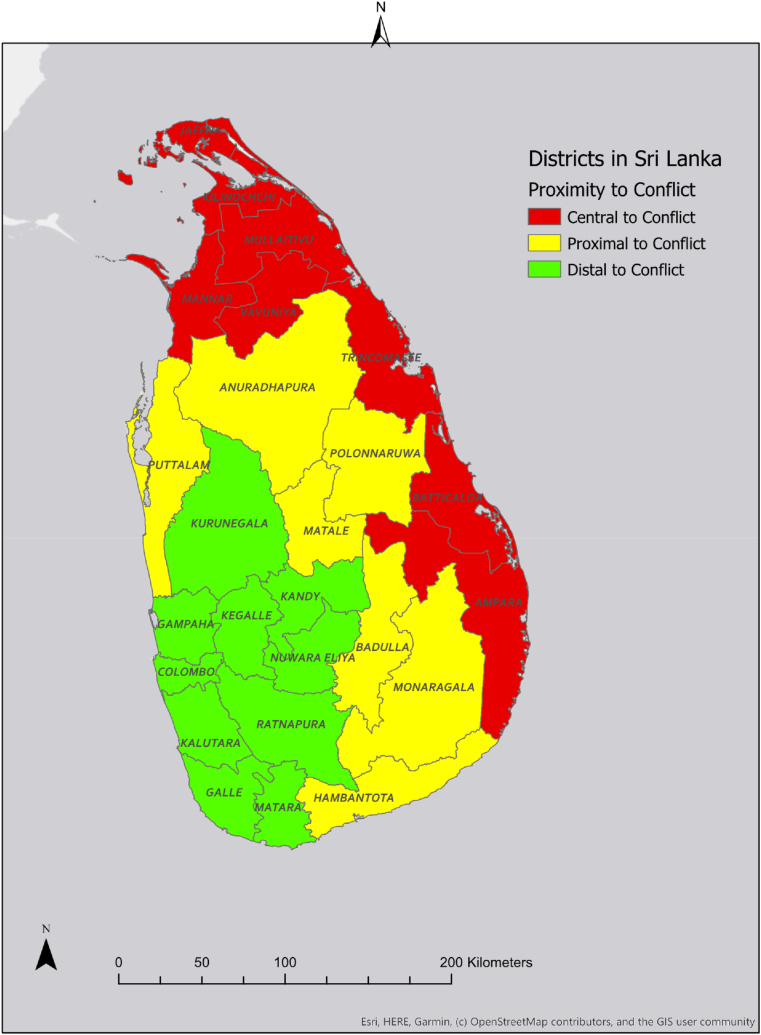
Map of Sri Lankan districts. Figure reproduced from Fonseka, R. W., Mcdougal, L., Raj, A., Reed, E., Lundgren, R., Urada, L., & Silverman, J. G. (2022b). Does Proximity to Conflict Zones Moderate Associations Between Girl Child Marriage, Intimate Partner Violence, and Contraception in Postconflict Sri Lanka? *Advances in Global Health*, *1*(1), 1–14. https://doi.org/10.1525/agh.2022.1539582.

We considered proximity to conflict areas as a potential moderator of the relationships between maternal child marriage and past year IPV and stunting. This variable was defined as having three levels: central, proximal, and distal. Dyads assigned to the “central” category reported residing in either the Northern or Eastern provinces of Sri Lanka, where the majority of the armed conflict occurred during the civil war. Central districts were Ampara, Batticaloa, Jaffna, Kilinochchi, Mannar, Mullaitivu, Trincomalee, and Vavuniya. Participants assigned to the “proximal” category reported residing in one of the seven districts of Sri Lanka outside of the Northern and Eastern provinces that shared a border with one of both of these provinces. Proximal districts were Anuradhapura, Badulla, Hambantota, Matale, Monaragala, Polonnaruwa, and Puttalam. Finally, participants assigned to the “distal” category resided in districts that were neither in nor bordering the Northern and Eastern provinces of the country. The “distal” districts were defined as Colombo, Galle, Gampaha, Kalutara, Kandy, Kegalle, Kurunegala, Nuwara Eliya, Matara, and Ratnapura. A map of Sri Lanka showing the geographic distribution of proximity to conflict is included as Fig. 1.

#### 2.2.4 Covariates

We included as covariates variables known or hypothesized to influence the relationship between independent variables of interest and stunting. We considered the child's sex (male or female), age in months (0-59), and birth order (1, 2, or 3 or above). The maternal variables we considered were height (short: ≤145 cm, average: >145 cm and ≤155 cm, or tall: > 155 cm) (Abeywickrama & Anuranga, 2020), age (18–49 years), education (primary, secondary, or higher than secondary), and age difference between mother and her partner (in years). At the household level, we considered household wealth quintile, religion (Buddhism, Hinduism, Islam, or other religion), and ethnicity (Sinhala, Sri Lankan Tamil, or other). Beyond individual- and household-level variables, we also included covariates at the geographic level to control for unmeasured variation between different settings, considering whether the mother and child lived in an urban setting (yes or no), and their district of residence (one of 25 administrative divisions across Sri Lanka).

### 2.3 Statistical analysis

All statistical analyses were conducted using R software version 3.6.3 (Chambers, 2008). Estimates were adjusted for complex survey design and participant-level weights using the “survey” package (Lumley, 2020) in order to calculate population-representative measures. We first assessed the distributions of maternal child marriage, all three forms of past year IPV, proximity to conflict, stunting, and all considered covariates in the sample. Next, we tested if the pairwise associations between each of the variables of interest and stunting were statistically significant. We used chi-squared tests to assess the pairwise distributions of each variable with the categorical outcome of stunting after reducing all variables to categories (ordinal or nominal). In preparation for our multivariable model, we assessed all variables for multicollinearity by calculating their variance inflation factor (VIF), and variables with VIF values above 5 were examined for overlap in distribution with other variables in the model. We kept in the analysis all covariates which could be retained with variance inflation factor (VIF) values less than 5, eliminating only religion.

We calculated the unadjusted change in odds of stunting (compared to no stunting) for each of our variables of interest. We then created a multivariable logistic regression model inclusive of all variables of interest as well as covariates to test the adjusted effects of each of our variables of interest on stunting. In order to control for all covariates which were marginally associated with stunting across our mother-child dyads, we included in this model all of the considered covariates that were associated with stunting at p ≤ 0.2 in pairwise distributions. Next, we examined the role of proximity to conflict as a potential moderator of the relationships between maternal child marriage, maternal past year IPV and stunting by creating four separate interaction terms that combined proximity to conflict with maternal child marriage and with each form of maternal past year IPV (Baron & Kenny, 1986). We assessed the significance of these interaction terms in a combined logistic regression model for stunting that also contained maternal child marriage, the maternal past year IPV variables, proximity to conflict, and all covariates included in the previous full regression model. Finally, we examined relationships between variables with interaction effects of p ≤ 0.1 and stunting in adjusted regression models stratified by proximity to conflict in order to understand how relationships between the independent variables and stunting were impacted by differing proximity to conflict.

## 3 Results

Descriptive characteristics of the sample and their distributions by stunting are summarized in Table 1. Over one in ten (13%) children were stunted. Nearly one in six (15%) mothers had married before the age of 18. Maternal experiences of IPV in the past year varied, with 2% reporting having experienced past year sexual IPV and 13% having experienced past year emotional IPV. The majority of households (64%) were in districts which were distal to conflict. One in four children (26%) had two or more older siblings. Mothers were mainly between 145 and 155 cm tall, older than 29, had attended secondary education, and were younger than their male partner. A majority of households were also Buddhist (72%), Sinhala (77%), and did not live in an urban setting (85%).

**Table 1:** Demographic details of youngest singleton-birth children under 5 years old of currently partnered women age 18–49 who participated in the 2016 Sri Lanka DHS Domestic Violence module by stunting (N = 4941) (Continued).

Characteristic	Stunting						
	Total		No		Yes		Chi-squared
	n	%	n	%^a^	n	%^a^	p-value
***Total***	4941	100%	4263	87%	678	13%	–
***Independent Variables***							
**Maternal Child Marriage**							
**No**	4249	85%	3680	87%	569	13%	0.11
**Yes**	692	15%	583	85%	109	15%	
**Maternal Past Year Sexual IPV**							
**No**	4826	98%	4172	87%	654	13%	0.08
**Yes**	115	2%	91	81%	24	19%	
**Maternal Past Year Physical IPV**							
**No**	4489	91%	3891	87%	598	13%	0.09
**Yes**	452	9%	372	84%	80	16%	
**Maternal Past Year Emotional IPV**							
**No**	4289	87%	3727	87%	562	13%	0.07
**Yes**	652	13%	536	85%	116	15%	
***Potential Moderator***							
**Proximity to Conflict**							
**Distal**	2690	64%	2314	86%	376	14%	0.08
**Proximal**	1121	23%	988	89%	133	11%	
**Central**	1130	13%	961	86%	169	14%	
***Demographic Characteristics***							
**Sex**							
**Male**	2571	52%	2208	87%	363	13%	0.59
**Female**	2370	48%	2055	87%	315	13%	
**Age in months**^b^							
**0**–**11**	967	20%	859	90%	108	10%	<0.01*
**12**–**35**	2174	44%	1815	84%	359	16%	
**36**–**59**	1800	36%	1589	88%	211	12%	
**Birth order**							
**1**	1649	35%	1446	88%	203	12%	0.16
**2**	1929	39%	1667	86%	262	14%	
**3 or above**	1363	26%	1150	86%	213	14%	
**Maternal Height**							
**≤145 cm**	362	8%	240	68%	122	32%	<0.01*
**>145 cm and** ≤ **155 cm**	2758	57%	2329	85%	429	15%	
**>155 cm**	1821	35%	1694	93%	127	7%	
**Maternal Age**^b^							
**18–29 years**	1843	23%	1572	86%	271	14%	0.14
**30–39 years**	2690	42%	2337	88%	353	12%	
**40–49 years**	408	35%	354	86%	54	14%	
**Maternal Education**							
**Primary (01–05) or less**	259	8%	198	77%	61	23%	<0.01*
**Secondary (6-10)**	3295	67%	2803	86%	492	14%	
**Higher than Secondary**	1387	26%	1262	91%	125	9%	
**Age difference between mother and partner**^b^							
**no difference or mother is older**	952	17%	818	87%	134	13%	0.49
**1–5 years**	2492	49%	2158	87%	334	13%	
**6–10 years**	1238	27%	1071	86%	167	14%	
**over 10 years**	259	6%	216	84%	43	16%	
**Household wealth quintile**							
**Lowest**	1212	18%	966	80%	246	20%	<0.01*
**Second**	1030	20%	867	84%	163	16%	
**Middle**	956	21%	850	89%	106	11%	
**Fourth**	936	21%	836	89%	100	11%	
**Highest**	807	20%	744	92%	63	8%	
**Religion**							
**Buddhism**	3121	72%	2742	88%	379	12%	<0.01*
**Hinduism**	842	11%	686	82%	156	18%	
**Islam**	517	9%	440	86%	77	14%	
**All other religions**	461	8%	395	87%	66	13%	
**Ethnicity**							
**Sinhala**	3337	77%	2938	88%	399	12%	<0.01*
**Sri Lankan Tamil**	994	12%	820	83%	174	17%	
**Other ethnicities**^c^	610	11%	505	84%	105	16%	
**Urban Setting**							
**No**	4161	85%	3582	87%	579	13%	0.38
**Yes**	780	15%	681	88%	99	12%	
**District**^d^							<0.01*

Frequency (n) values are unweighted, while percent values and p-values are weighted according to the survey's complex sampling design.

*p ≤ 0.05.

a Cross-tabulated weighted percent values are calculated within rows.

b Age in months, maternal age, and age difference between mother and partner are presented in Table 1, Table 2 as categorical to facilitate cross-tabulations, but were included in regression models as continuous variables.

c Other ethnicities include Muslim, Malay, Indian Tamil, Burgher, and other.

d A full list of districts is omitted due to length (see Appendix Table 1).

Children's stunting varied significantly in pairwise unadjusted associations with many characteristics (measured by a chi-squared test of distribution, p value less than or equal to 0.05). Maternal child marriage was not significantly associated with stunting (p = 0.11), while all three forms of maternal past year IPV showed marginal positive associations with stunting (p ≤ 0.1). Children in districts proximal to conflict appeared marginally less likely to experience stunting (p = 0.08) compared to children in areas distal or central to conflict. Covariates significantly associated with stunting (p ≤ 0.05) in chi-squared pairwise analyses were child's age, maternal height, maternal education, household wealth, religion, ethnicity, and district of residence.

The change in the odds of stunting (odds ratio) associated with maternal child marriage and each form of maternal past year IPV can be found in Table 2. Neither maternal child marriage nor any form of past year IPV were significantly associated with changes in the odds of stunting in the national adjusted regression model. In contrast, associations between proximity to conflict and stunting were present in the adjusted model, with significantly lower odds of stunting among children in districts that were proximal (adjusted odds ratio/aOR: 0.43, 95% confidence interval/CI: 0.22–0.82) and central (aOR: 0.53; CI: 0.29–0.98) compared to children in areas distal to conflict.

**Table 2:** Logistic regression comparing stunting to no stunting among youngest singleton-birth children under 5 years old of currently partnered women age 18–49 who participated in the 2016 Sri Lanka DHS Domestic Violence module (N = 4941).

Variables of interest	Stunting (compared to no stunting)					
	Unadjusted OR			Adjusted OR^a^		
	OR	95% CI	p-value	aOR	95% CI	p-value
***Independent Variables***						
**Maternal Child Marriage**						
**No**	ref	ref	Ref	ref	ref	ref
**Yes**	1.21	0.96, 1.52	0.11	0.95	0.70, 1.28	0.73
**Maternal Past Year Sexual IPV**						
**No**	ref	ref	Ref	ref	ref	ref
**Yes**	1.63	0.94, 2.81	0.08	1.17	0.64, 2.14	0.60
**Maternal Past Year Physical IPV**						
**No**	ref	ref	Ref	ref	ref	ref
**Yes**	1.29	0.96, 1.72	0.09	0.95	0.67, 1.34	0.76
**Maternal Past Year Emotional IPV**						
**No**	ref	ref	Ref	ref	ref	ref
**Yes**	1.26	0.98, 1.61	0.07	1.04	0.76, 1.42	0.80
***Potential Moderator***						
**Proximity to Conflict**						
**Distal**	ref	ref	Ref	ref	ref	ref
**Proximal**	0.81	0.66, 1.00	0.05	0.43	0.22, 0.82	0.01*
**Central**	1.04	0.84, 1.29	0.71	0.53	0.29, 0.98	0.04*

OR=Odds Ratio, aOR = adjusted Odds Ratio, CI = Confidence Interval.

*p ≤ 0.05.

a Multivariate model included as covariates age in months, birth order, maternal height, maternal age, maternal education, household wealth quintile, ethnicity, and district.

Results from the interaction analysis are shown in Table 3. There was a significant interaction between maternal past year sexual IPV and centrality to conflict (aOR: 0.15, CI: 0.04–0.53), suggesting that proximity to conflict moderates the relationship between maternal past year sexual IPV and stunting. Additionally, emotional IPV had a marginal (p ≤ 0.1) interaction with proximity to conflict in both the proximal (aOR: 0.41, CI: 0.15, 1.11) and central to conflict (aOR: 1.78, CI: 0.91, 3.47) categories.

**Table 3:** Interaction effects of proximity to conflict on associations between maternal child marriage, past year IPV and stunting among youngest singleton-birth children under 5 years old of currently partnered women age 18–49 who participated in the 2016 Sri Lanka DHS Domestic Violence module (N = 4941).

Variable of interest	Stunting (compared to no stunting)		
	Adjusted OR^a^		
	aOR	95% CI	p-value
Proximity to Conflict*Maternal Child Marriage Interaction			
Proximal*Maternal Child Marriage	0.96	0.51, 1.81	0.90
Central*Maternal Child Marriage	1.20	0.63, 2.29	0.58
Proximity to Conflict*Sexual IPV Interaction			
Proximal*Past Year Sexual IPV	0.56	0.09, 3.39	0.58
Central*Past Year Sexual IPV	0.15	0.04, 0.53	<0.01*
Proximity to Conflict*Physical IPV Interaction			
Proximal*Past Year Physical IPV	1.14	0.43, 3.02	0.78
Central*Past Year Physical IPV	1.32	0.61, 2.84	0.48
Proximity to Conflict*Emotional IPV Interaction			
Proximal*Past Year Emotional IPV	0.41	0.15, 1.11	0.08
Central*Past Year Emotional IPV	1.78	0.91, 3.47	0.09

aOR = adjusted Odds Ratio, CI = Confidence Interval.

*p ≤ 0.05.

a Multivariate model included maternal child marriage; maternal past year sexual, emotional, and physical IPV; and proximity to conflict; as well the following covariates: age in months, birth order, maternal height, maternal age, maternal education, household wealth quintile, ethnicity, and district.

Results of the stratified analysis of the impact of past year sexual and emotional IPV on stunting by districts that were distal (n = 2690), proximal (n = 1121), and central (n = 1130) to conflict are shown in Table 4. Maternal past year sexual IPV was significantly associated with increased odds of stunting in areas distal to conflict (aOR: 2.71, CI: 1.16–6.35). Maternal past year emotional IPV was significantly associated with decreased odds of stunting in districts proximal to conflict (aOR: 0.42, CI: 0.18–0.96), and increased odds of stunting in districts central to conflict (aOR: 1.80, CI: 1.13–2.89).

**Table 4:** Stratified logistic regressions across proximity to conflict comparing odds of stunting to no stunting among youngest singleton-birth children under 5 years old of currently partnered women age 18–49 who participated in the 2016 Sri Lanka DHS Domestic Violence module (N = 4941).

*Proximity to Conflict*	Stunting (compared to no stunting)								
	*Distal (n* = *2690)*			*Proximal (n* = *1121)*			*Central (n* = *1130)*		
Variable	aOR	95% CI	p-value	aOR	95% CI	p-value	aOR	95% CI	p-value
**Maternal Past Year Sexual IPV**									
**No**	Ref	ref	ref	ref	ref	ref	ref	ref	ref
**Yes**	2.71	1.16, 6.35	0.02*	1.42	0.30, 6.73	0.66	0.48	0.19, 1.23	0.13
**Maternal Past Year Emotional IPV**									
**No**	Ref	ref	ref	ref	ref	ref	ref	ref	ref
**Yes**	0.90	0.59, 1.36	0.61	0.42	0.18, 0.96	0.04*	1.80	1.13, 2.89	0.01*

aOR = adjusted Odds Ratio, CI = Confidence Interval.

*: p ≤ 0.05.

Multivariate models included as covariates age in months, birth order, maternal height, maternal age, maternal education, household wealth quintile, ethnicity, and district. Models did not include maternal child marriage or maternal past year physical IPV, due to lack of interaction with proximity to conflict.

## 4 Discussion

Our study looked at the impacts of maternal child marriage and IPV on stunting in Sri Lanka, and the potential role of proximity to conflict as a moderator of these relationships. In adjusted models across the country, neither maternal child marriage nor any form of maternal past year IPV appeared to be associated with stunting, while children living in districts proximal and central to conflict were less likely to be stunted than in children distal to conflict. Proximity to conflict significantly moderated relationships between maternal sexual and emotional IPV and stunting, with a further stratified analysis revealing that sexual IPV was associated with increased stunting only in districts distal to conflict, while emotional IPV was associated with increases in stunting in areas central to conflict but decreased stunting in areas proximal to conflict. We did not find associations between maternal child marriage or past year physical IPV and stunting. Our findings suggest that Sri Lankan children born to mothers contending with IPV may face a heightened vulnerability to stunting that varies (is moderated) by their proximity to conflict.

We did not find country-wide associations between maternal child marriage or any form of maternal IPV and stunting. However, this lack of association at the country level did not preclude the possibility of moderated associations between the independent variables and stunting that differed by proximity to conflict. This investigation was further supported by our finding that proximity to conflict was associated with stunting, and previous research had found conflict to also be associated with child marriage and IPV (Bandara et al., 2021; Gottschalk, 2007; Kelly et al., 2018; Mourtada et al., 2017; Raj, 2010). In our full adjusted regression model, children living in districts proximal and central to conflict had decreased odds of stunting for compared to those living distal to conflict. One possible reason for these findings might be the positive impact and differential implementation of humanitarian and post-conflict aid programs focused on childhood nutrition, which are more likely to be implemented in areas central and proximal to conflict zones within a country. This possible explanation is supported by a 2016 study of food insecurity in the central to conflict Jaffna district, which found that food insecurity among households included in the study was 10 percent, compared to a prevalence rate of 76 percent in the distal district of Kurunegala (Kandeepan et al., 2016). Evidence on the effectiveness of humanitarian interventions to improve health is inconclusive (Blanchet et al., 2017), and our finding of reduced stunting in areas most affected by conflict in post-conflict Sri Lanka contributes to this literature. Another possible explanation for decreased stunting in the proximal and central districts might be increased child mortality in these areas compared to distal districts. Higher mortality among children under five might result in a population of surviving children who are less at risk for stunting. This potential explanation is supported by a 2016 analysis conducted by the Sri Lankan Department of Census and Statistics which found that the central districts Kilinochchi, Trincomalee, and Mullaitivu and proximal district Puttalam had the four highest rates of under-five mortality in Sri Lanka (Department of Census and Statistics, 2017).

We found that proximity to conflict moderated the associations linking maternal past year sexual and emotional IPV with stunting. In areas distal to conflict, maternal past year sexual IPV was associated with increased stunting, an association that has also been found in other countries (Sobkoviak et al., 2012; Ziaei et al., 2014). However, this relationship did not exist in areas proximal or central to conflict, suggesting that the practices that could link sexual IPV and stunting in these areas, such as variation in nutritional practices including breastfeeding, may be impacted by enduring effects of the conflict. Maternal past year emotional IPV was associated with increased stunting in districts central to conflict, and decreased stunting in districts proximal to conflict, with no association in districts distal to conflict. These findings highlight the differences between children and families residing in districts which are proximal to rather than central to conflict – perhaps protective factors such as increased child welfare services exist in the proximal districts which prevent maternal emotional IPV from having the negative consequences to child health that it is associated with in districts central to conflict. One study of Sri Lankan health infrastructure found differential health services utilization between proximal and central districts (Johnson, 2017), further supporting the idea that children in proximal districts may have access to resources to prevent maternal emotional IPV from resulting in poor nutrition and stunting in ways that children in central districts do not. Furthermore, the differential associations found between stunting and each form of IPV highlights the difference in these experiences as risk factors and the importance of studying the impact of IPV in its various forms when assessing its impact on health.

The lack of association between stunting and maternal child marriage in our study sample contradicts research from other settings linking maternal child marriage to stunting and child malnutrition. For example, one study of nearly 40,000 mother-child pairs across Sub-Saharan Africa found that the odds of stunting were 29% higher for children born to women who married before 18 compared to those whose mothers married later (Efevbera et al., 2017), and a study in India found that malnutrition was higher in children born to mothers married as minors (Raj et al., 2010). One possible explanation for this discrepancy is the relatively high age of mothers in this study's sample – over half of the Sri Lankan mothers included in this study were older than 29 years old. The associations found in other countries between maternal child marriage and child malnutrition may be facilitated by early childbearing, as explained in research that shows associations between infant mortality and young maternal age (Raj et al., 2014). In the case of this sample, although many mothers were married before age 18, they may have given birth to the child included in this study many years into adulthood.

This study had multiple limitations. We did not have data on paternal height, which could also influence stunting. By using binary IPV variables, we were not able to assess the differential impact of varying frequency of IPV. The DHS only collected data on past year experience of IPV, therefore we were also unable to analyze the impacts of lifetime IPV as compared to past year. To avoid overadjustment, we did not include variables that might be on the causal pathway between our maternal risk factors of interest and stunting, such as low birthweight and exclusive breastfeeding. Future studies of the impact of these factors on stunting in Sri Lanka would be very valuable. The survey did not collect complete information on women's location over time, only collecting information on the most recent location that women had moved from. Therefore, we cannot make conclusions about the direct impact of the conflict on survey participants, which may have varied greatly based on their location over time. Additionally, a district's geographic location was not the only determinant of its exposure to conflict-related violence; for example, Colombo district, which is categorized as distal to conflict, experienced several attacks during the war which exposed its residents to violence and trauma. For these reasons, our “proximity to conflict” variable must be interpreted in a post-conflict and cross-sectional context, seven years after the end of the Sri Lankan civil war, and as a geographic, not experiential, variable. We suggest that future DHS implementations in Sri Lanka collect anonymized geolocation data and ask all participants about their migration histories so that the impact of lifetime exposure to conflict can be examined in future analyses. In this analysis, we excluded all participants below the age of 18 in order to correctly measure our independent variable of maternal child marriage. However, early childbearing is a risk factor for child malnutrition and other negative health consequences (Raj, 2010), therefore further research which focuses primarily on mothers below the age of 18 in Sri Lanka would be a valuable contribution to the scientific literature.

## 5 Conclusions

Children in districts proximal and central to conflict were less likely to be stunted compared to children in districts distal to conflict, suggesting the existence of additional (possibly humanitarian) nutritional resources for children in areas impacted heavily by conflict. Proximity to conflict moderated the impacts of maternal past year sexual and emotional IPV on stunting, with maternal sexual IPV increasing the odds of stunting in districts distal to conflict and emotional IPV increasing odds of stunting in districts central to conflict. Conversely, maternal past year emotional IPV was associated with decreased odds of stunting in proximal districts, suggesting additional resources for women experiencing IPV or children at risk of malnutrition in those areas. Conducted among children born after the end of the Sri Lankan civil war in 2009, this study suggests that armed conflict, and its ensuing humanitarian interventions, can have lasting and intergenerational impacts on health. Programs to address stunting in Sri Lanka should consider the role of maternal IPV and take into account the varying contexts and existing resources of areas of the country differentially exposed to the conflict. With targeted interventions, disparities in child nutrition can be addressed and the impacts of conflict can be prevented from harming future generations.

## Data availability statement

Data from the 2016 Sri Lankan Demographic and Health Survey can be requested from the Sri Lankan Office of the Census. Information on requesting this data can be found at the Department’s website: http://www.statistics.gov.lk/.

## Funding statement

This work was supported by the 10.13039/100000865Bill & Melinda Gates Foundation, Seattle, WA [grant number OPP1179208; PI: Raj]. The funding source had no involvement in the study design, collection, analysis and interpretation of data, writing of the report, or in the decision to submit the article for publication.

## Ethical approval

Ethical approval for this research was obtained from the University of California, San Diego Institutional Review Board (Project number #191418XX).

## Author contributions section

Ruvani W. Fonseka: Conceptualization; Data curation; Formal analysis; Investigation; Methodology; Software; Visualization; Writing - original draft; Writing - review & editing.

Jay G. Silverman: Conceptualization; Funding acquisition; Methodology; Supervision; Validation; Writing - review & editing.

Lotus McDougal: Conceptualization; Funding acquisition; Methodology; Supervision; Validation; Writing - review & editing.

Anita Raj: Conceptualization; Funding acquisition; Methodology; Supervision; Validation; Writing - review & editing.

Elizabeth Reed: Conceptualization; Methodology; Supervision; Validation; Writing - review & editing.

Lianne Urada: Conceptualization; Methodology; Supervision; Validation; Writing - review & editing.

Rebecka Lundgren: Conceptualization; Methodology; Supervision; Validation; Writing - review & editing.

## Declaration of competing interest

The authors have no conflicts of interest to declare.

## Figures and Tables

**Table:** 

Characteristic	Stunting						
	Total		No		Yes		Chi-squared
	n	%	N	%^∧^	n	%^∧^	p-value
**District**							
***Distal to Conflict***							
**Colombo**	366	10%	313	86%	53	14%	<0.01*
**Galle**	226	5%	204	90%	22	10%	
**Gampaha**	367	11%	336	89%	31	11%	
**Kalutara**	226	6%	201	89%	25	11%	
**Kandy**	299	7%	235	79%	64	21%	
**Kegalle**	165	4%	139	83%	26	17%	
**Kurunegala**	386	8%	331	86%	55	14%	
**Matara**	208	4%	185	89%	23	11%	
**Nuwara Eliya**	169	3%	121	72%	48	28%	
**Ratnapura**	278	6%	249	90%	29	10%	
***Proximal to Conflict***							
**Anuradhapura**	160	4%	127	80%	33	20%	
**Badulla**	216	5%	189	88%	27	12%	
**Hambantota**	142	3%	129	91%	13	9%	
**Matale**	109	2%	94	89%	15	11%	
**Monaragala**	164	3%	148	89%	16	11%	
**Polonnaruwa**	143	2%	130	92%	13	8%	
**Puttalam**	187	4%	171	91%	16	9%	
***Central to Conflict***							
**Ampara**	158	3%	139	87%	19	13%	
**Batticaloa**	126	2%	115	92%	11	8%	
**Jaffna**	227	4%	190	83%	37	17%	
**Kilinochchi**	116	0%	93	80%	23	20%	
**Mannar**	138	0%	113	81%	25	19%	
**Mullaitivu**	99	0%	86	87%	13	13%	
**Trincomalee**	145	2%	124	86%	21	14%	
**Vavuniya**	121	1%	101	86%	20	14%	

Frequency (n) values are unweighted, while percent values and p-values are weighted according to the survey's complex sampling design.

*p < 0.05.

^∧^cross-tabulated weighted percent values are calculated within rows.

## References

[bib1] Abeywickrama G., Anuranga C. (2020). A decomposition analysis of inequalities in low birth weight in Sri Lanka: Findings from the demographic and health survey- 2016. Ceylon Medical Journal.

[bib2] Bandara P., Knipe D., Munasinghe S., Rajapakse T., Page A. (2021).

[bib3] Baron R.M., Kenny D.A. (1986). The moderator-mediator variable distinction in social psychological research. Conceptual, strategic, and statistical considerations. Journal of Personality and Social Psychology.

[bib4] Blanchet K., Ramesh A., Frison S., Warren E., Hossain M., Smith J., Knight A., Post N., Lewis C., Woodward A., Dahab M., Ruby A., Sistenich V., Pantuliano S., Roberts B. (2017). Evidence on public health interventions in humanitarian crises. The Lancet.

[bib5] Boerma T., Tappis H., Saad-Haddad G., Das J., Melesse D.Y., DeJong J., Spiegel P., Black R., Victora C., Bhutta Z.A., Barros A.J.D. (2019). Armed conflicts and national trends in reproductive, maternal, newborn and child health in sub-saharan Africa: What can national health surveys tell us?. BMJ Global Health.

[bib6] Chambers J. (2008).

[bib7] Department of Census and Statistics (2017).

[bib8] Efevbera Y., Bhabha J., Farmer P.E., Fink G. (2017). Girl child marriage as a risk factor for early childhood development and stunting. Social Science & Medicine.

[bib9] Ellsberg M., Heise L. (2005). https://www.who.int/reproductivehealth/publications/violence/9241546476/en/.

[bib10] Fokus Women (2015). https://docplayer.net/24861265-Post-war-trends-in-child-marriage-sri-lanka.html.

[bib11] Fonseka R.W., Mcdougal L., Raj A., Reed E., Lundgren R., Urada L., Silverman J.G. (2022). A mediation analysis of the role of girl child marriage in the relationship between proximity to conflict and past - year intimate partner violence in post - conflict Sri Lanka. Conflict and Health.

[bib12] Fonseka R.W., Mcdougal L., Raj A., Reed E., Lundgren R., Urada L., Silverman J.G. (2022). Does proximity to conflict zones moderate associations between girl child marriage , intimate partner violence , and contraception in postconflict Sri Lanka. Advances in Global Health.

[bib13] Gottschalk N. (2007). Uganda: Early marriage as a form of sexual violence. Forced Migration Review.

[bib14] Jayasekara R.S., Schultz T. (2007). Health status, trends, and issues in Sri Lanka. Nursing and Health Sciences.

[bib15] Johnson S.A. (2017). The Cost of War on Public Health: An exploratory method for understanding the impact of conflict on public health in Sri Lanka. PLoS One.

[bib16] Kandeepan K., Balakumar S., Arasaratnam V. (2016). Nutritional status and food insecurity among the children in northern Sri Lanka. Procedia Food Science.

[bib17] Kannaujiya A.K., Kumar K., Upadhyay A.K., McDougal L., Raj A., Singh A. (2020). Short interpregnancy interval and low birth weight births in India: Evidence from National Family Health Survey 2015-16. SSM - Population Health.

[bib18] Kelly J.T.D., Colantuoni E., Robinson C., Decker M.R. (2018). From the battlefield to the bedroom: A multilevel analysis of the links between political conflict and intimate partner violence in Liberia. BMJ Global Health.

[bib19] Kottegoda S., Samuel K., Emmanuel S. (2008). Reproductive health concerns in six conflict-affected areas of Sri Lanka. Reproductive Health Matters.

[bib20] Loaiza E., Wong S. (2012). Marrying too young: End child marriage. United Nations Population Fund UNFPA.

[bib21] Lumley T. (2020). https://cran.r-project.org/web/packages/survey/survey.pdf.

[bib22] McCall C. (2016). Sri Lanka's war wounds run deep. The Lancet.

[bib23] Minoiu C., Shemyakina O.N. (2014). Armed conflict, household victimization, and child health in CÔte d'Ivoire. Journal of Development Economics.

[bib24] Misch E.S., Yount K.M. (2014). Intimate partner violence and breastfeeding in Africa. Maternal and Child Health Journal.

[bib25] Mourtada R., Schlecht J., Dejong J. (2017). A qualitative study exploring child marriage practices among Syrian conflict-affected populations in Lebanon. Conflict and Health.

[bib26] Naotunna N.P.G.C.R., Dayarathna M., Maheshi H., Amarasinghe G.S., Kithmini V.S., Rathnayaka M., Premachandra L., Premarathna N., Rajasinghe P.C., Wijewardana G., Agampodi T.C., Agampodi S.B. (2017). Nutritional status among primary school children in rural Sri Lanka; A public health challenge for a country with high child health standards. BMC Public Health.

[bib27] O’hare B.A.M., Southall D.P. (2007). First do no harm: The impact of recent armed conflict on maternal and child health in Sub-Saharan Africa. Journal of the Royal Society of Medicine.

[bib28] de Onis M., Branca F. (2016). Childhood stunting: A global perspective. Maternal and Child Nutrition.

[bib29] Raj A. (2010). When the mother is a child: The impact of child marriage on the health and human rights of girls. Archives of Disease in Childhood.

[bib30] Raj A., McDougal L., Rusch M.L.A.A. (2014).

[bib31] Raj A., Saggurti N., Winter M., Labonte A., Decker M.R., Balaiah D., Silverman J.G. (2010). The effect of maternal child marriage on morbidity and mortality of children under 5 in India: Cross sectional study of a nationally representative sample. BMJ.

[bib32] Rannan-Eliya R.P., Hossain S.M.M., Anuranga C., Wickramasinghe R., Jayatissa R., Abeykoon A.T. (2013). Trends and determinants of childhood stunting and underweight in Sri Lanka. Ceylon Medical Journal.

[bib33] Sengupta S. (2009, May 19). https://www.nytimes.com/2009/05/20/world/asia/20lanka.html.

[bib34] Sobkoviak R.M., Yount K.M., Halim N. (2012). Domestic violence and child nutrition in Liberia. Social Science & Medicine.

[bib35] Wondimagegn Z.T. (2014). Magnitude and determinants of stunting among children in Africa: A systematic review. Current Research in Nutrition and Food Science.

[bib36] World Health Organization (2013).

[bib37] World Health Organization (2015). https://www.who.int/healthinfo/indicators/2015/en/.

[bib38] Ziaei S., Naved R.T., Ekström E.C. (2014). Women's exposure to intimate partner violence and child malnutrition: Findings from demographic and health surveys in Bangladesh. Maternal and Child Nutrition.

